# Loot

**Published:** 1869-05-01

**Authors:** 


### LOOT.

### *Poisoning by Atropia.*

The *Am. Jour. of Pharmacy* reports the result of the trial of Mademoiselle J., at Geneva, Switzerland, for poisoning seven persons with atropia, administered to them as *kirschenswasser*, while they were under her care as nurse. Notwithstanding the plea of insanity, for which the circumstances furnished strong ground, she was sentenced to penal servitude.

### *Extirpation of Lachrymal Gland.*

Dr. A. D. Williams, of Cincinnati, has successfully extirpated the lachrymal gland, in a case of imperforate canaliculi, and recommends the operation in such cases, but opposes the recommendation of Lawrence and others to remove the gland in ordinary cases of obstructed nasal duct.—*Cinn- Lancet and Observer.*

### *Spurious Vaccination.*

Dr. B. Rormer, of Kanawha Salings, W. Virginia, reports to the same journal a synopsis of four hundred and fifty-seven cases of spurious vaccinnation; constituting, perhaps, the most comprehenslve condensed

### *Opium Culture.*

Mr. W. P. Creecy, of Vicksburg, Miss., reports to the same journal the failure of his experiments in the culture of the poppy, but suggests a continuation of the experiments in the South.

---

NOTE.—There is no doubt of the practicability of cultivating Opium in some portions of the South, as the writer has tested experimentally in Florida, by using the inspissated juice of the common poppy, which was found to possess similar properties to the opium of commerce. The experiments were, of course, upon a very small scale. Repeated attempts to induce germination in imported seed failed, inducing the belief in the correctness of the commonly received opinion that the capsules were baked before exportation, in order to prevent germination. W. H.

***Anatomy.***

The Legislature of the State of Maine has indefinitely postponed the bill for the legalization of the study of anatomy. It is one of the most remarkakle inconsistencies of an enlightened (?) age, that society should require the possession of certain knowledge by certain of its members, and withhold, under penalty, the only means whereby such knowledge can be acquired.

The same journal contains a formula for the preparation of carbolic acid plaster, to be used as an antiseptic dressing for wounds. We give it viz.:

Olive oil, 12 parts (by measure).
Litharge, (finely ground) 12 parts (by weight).
Beeswax, 3 parts (by weight).
Crystallized carbolic acid, 2½ parts (by weight).

Heat half the olive oil over a slow fire, then add the litharge gradually, stirring constantly until the mass becomes thick or a little stiff; then add the other half of the oil, stirring as before, till it becomes again thick. Then add the wax gradually, till the liquid again thickens. Remove from the fire, add the acid, stirring briskly till thoroughly mixed. Cover up close and set aside, to allow the residual litharge to settle; then pour off the fluid and spread upon calico to the proper thickness. If kept in a well-fitting tin cannister it will retain its virtues for any length of time. Another form of antiseptic plaster is the following:

Shellac, 3 parts.
Crystallized Carbolic Acid, 1 part.

Heat the lac, with about one-third of the carbolic acid, over a slow fire, till the lac is completely melted; then remove from the fire and add the remainder of the acid, and stir briskly till the ingredients are thoroughly mixed. Strain through muslin, and when sufficiently cooled spread to the thickness of one-fiftieth of an inch; after, brush over theplaster with a solution of gutta percha in a solution of bisulphide of carbon.

